# Safety and Clinical Outcomes of Antibiotic Therapy for Helicobacter pylori Eradication During Pregnancy in Hyperemesis Gravidarum Patients

**DOI:** 10.7759/cureus.94606

**Published:** 2025-10-14

**Authors:** Muhammad Rehan Mumtaz, Komal Kiran Galaria, Muhammad Abid, Aftab Ahmad Khan, Hamza Usman, Mahrukh Ehsan

**Affiliations:** 1 Internal Medicine, Aziz Bhatti Shaheed Teaching Hospital, Gujrat, PAK; 2 Internal Medicine, Jinnah Medical College Hospital, Karachi, PAK; 3 Internal Medicine, Tehsil Headquarter (THQ) Hospital Ahmedpur East, Bahawalpur, PAK; 4 Medical Oncology, Mater Hospital, Dublin, IRL; 5 Obstetrics and Gynecology, Peterborough City Hospital, Peterborough, GBR

**Keywords:** antibiotic therapy, efficacy, eradication, helicobacter pylori, hyperemesis gravidarum, pregnancy, safety

## Abstract

Introduction

Hyperemesis gravidarum (HG) is a severe form of nausea and vomiting during pregnancy, often leading to significant maternal and fetal complications. *Helicobacter pylori* (*H. pylori*) infection has been associated with HG, suggesting that eradication of the bacterium may alleviate symptoms. This study evaluates the safety and outcomes of antibiotic therapy for *H. pylori* eradication in pregnant women diagnosed with HG.

Objective

To assess the safety and outcomes of antibiotic regimens in eradicating *H. pylori* and improving clinical outcomes in pregnant patients with HG.

Methodology

A cross-sectional study involving 186 pregnant women diagnosed with HG and confirmed *H. pylori* infection was conducted at Family Health Hospital, Islamabad, from June 2023 to January 2024. Participants received a standardized antibiotic regimen safe for pregnancy. Data on eradication rates, symptom improvement, hospitalization duration, and maternal and fetal outcomes were collected and analyzed.

Results

The antibiotic therapy achieved an *H. pylori* eradication rate of 85% (158 patients). Significant improvement in HG symptoms was observed in 145 (78%) patients, while 41 (22%) reported no symptomatic benefit. The average hospitalization duration was reduced from 7 ± 2 days before treatment to 3 ± 1 days post-treatment (p<0.01). No serious adverse effects were reported. Fetal outcomes were reassuring: the mean birth weight was 3200 ± 500 g, gestational age at delivery averaged 39 ± 1.5 weeks, preterm deliveries occurred in nine (4.8%) cases, and low birth weight infants were recorded in seven (3.8%) cases; figures comparable to national averages.

Conclusion

Antibiotic therapy for *H. pylori* eradication is both safe and effective in managing HG during pregnancy. This treatment approach may offer a viable strategy to alleviate severe symptoms and improve maternal and fetal outcomes.

## Introduction

Hyperemesis gravidarum (HG) is known as one of the most challenging disorders a pregnant mother may be subjected to, which is manifested by excessive nausea and constant vomiting that lasts longer than the morning sickness many would-be mothers come to be acquainted with. Comprising 0.3 to 2 percent of all pregnancies, HG may cause significant maternal and fetal morbidities, which include dehydration, electrolyte disorders, and weight loss, and in severe conditions, malnutrition. The condition not only exposes the physical health of the mother, but it is also a psychologically and emotionally challenging condition that causes more stress, anxiety, and depression throughout the pregnancy [1,2].

The causes of HG are complex and multifactorial, therefore requiring a multiplicity of factors and interaction of hormonal, genetic, physiological, and environmental factors. The pathogenesis of HG has been associated with high levels of human chorionic gonadotropin (hCG) and estrogen, and hCG levels predict the degree of symptoms during the early stages of pregnancy. Moreover, the genetic backgrounds might make some women more prone to developing the HG, which implies the hereditary nature of the condition. The severity of HG may also be worsened by psychosocial stressors and prior mental health disorders, further reinforcing poor maternal health. Recent evidence suggests that there is a possibility of a relationship between Helicobacter pylori (H. pylori) infection and HG. H. pylori is a Gram-negative, microaerophilic bacterium that tends to colonize the digestive tract of humans and is a well-proven leading cause of chronic gastritis, peptic ulcer disease, and is linked to gastric cancer and mucosa-associated lymphoid tissue (MALT). Urease production aids the survival of the bacterium in the desolate acidic environment of the stomach because it neutralizes the gastric acid and enables it to attach onto the gastric mucosa to avoid being detected by the host immune system [3,4].

The assumption that H. pylori infection can cause HG is based on the fact that eradication of the bacteria can cause a substantial decrease in the symptoms of HG. The mechanisms suggested are the decrease of gastric inflammation, enhancement of gastric motility, and control of central nervous system pathways participating in reflexes of nausea and vomiting. Delayed gastric emptying and gastric hyperinflammation, which are possible sequelae of chronic H. pylori infection, are believed to contribute to the manifestations of HG through a self-cycling process of nausea and repeated vomiting, triggering an upsurge of the illness. Regardless of such promising correlations, the safety and effectiveness of antibiotic treatment for H. pylori eradication during pregnancy is a topic of research. Pregnant women pose a special population in clinical studies because interventions carried out might affect both the pregnant woman and the unborn child due to the associated ethical concerns and risk to both parties. Since antibiotics may actually be of some advantage in the clearing of H. pylori as well as in the improvement of the symptoms of HG, such a therapy will have to be considered critically so that no unnecessary risks are brought up that may cause the development of antibiotic resistance, or rather negative reproductive ventures. Past research has given inconsistent findings on the role of H. pylori in HG. There is some research evidence to suggest that H. pylori infection is significantly associated with HG severity, and some research has demonstrated no such relationship [5,6]. These inconsistencies are because of variation in study design, sample size, way of diagnosing H. pylori infection, and also the antibiotic regimen given in the eradication. Moreover, there could be a cultural and socioeconomic impact on the occurrence of H. pylori infection as well as the manifestation of HG, contributing to the necessity of local research affecting evidence-based clinical practice. This paper tries to fill the knowledge gap by systematically reviewing the safety and effectiveness of the regimen of antibiotics that are specially designed to be administered to pregnant women who have HG and are infected with H. pylori. The study aims to find evidence-based advice regarding the inclusion of the H. pylori eradication strategies in the practice of HG prevention and management by evaluating clinical outcomes, maternal well-being, and fetal health indicators. The results are meant to be used in the formulation of clinical guidelines as well as the optimization of treatment regimens and, ultimately, enhancing the quality of care to pregnant women, who have this most difficult condition [7,8].

The main objective of the study is to assess the safety and outcomes of antibiotic regimens in eradicating H. pylori and improving clinical outcomes in pregnant patients with HG.

## Materials and methods

This prospective cross-sectional study was conducted at Family Health Hospital, Islamabad, from June 2023 to January 2024. A total of 186 pregnant women diagnosed with HG and confirmed H. pylori infection were enrolled. All participants received a 14-day pregnancy-safe triple therapy consisting of amoxicillin 1 g twice daily, clarithromycin 500 mg twice daily, and omeprazole 20 mg twice daily. Patients intolerant to clarithromycin were switched to amoxicillin + metronidazole + lansoprazole (each twice daily). Follow-up stool antigen testing at four weeks confirmed H. pylori eradication. In the study, non-invasive diagnostic methods were used due to pregnancy safety considerations. Specifically, the stool H. pylori antigen test and the urea breath test were employed to confirm infection. Inclusion criteria included singleton pregnancy, gestational age between 8 and 20 weeks, and a diagnosis of HG based on standardized clinical criteria. Exclusion criteria were multiple gestations, known antibiotic allergies, contraindications to antibiotic therapy, and pre-existing gastrointestinal disorders. Inclusion and exclusion criteria are mentioned in Table 1.

**Table 1:** Inclusion and Exclusion Criteria

Inclusion Criteria	Exclusion Criteria
Singleton pregnancy	Multiple gestations (twins, triplets, etc.)
Gestational age between 8 and 20 weeks	Known allergies to antibiotics used in the regimen
Diagnosis of HG based on standardized clinical criteria	Contraindications to antibiotic therapy (e.g., liver or kidney dysfunction)
Confirmed *H. pylori* infection through diagnostic testing (stool *H. pylori* antigen test or a urea breath test)	Pre-existing gastrointestinal disorders (e.g., Crohn’s disease, ulcerative colitis)
Willingness to provide informed consent	Participants currently on other treatments for *H. pylori* eradication

Data collection

Data collection was meticulously carried out to ensure comprehensive and accurate information gathering from all 186 participants. Informed consent was obtained from all participants. The process began with structured interviews conducted by trained healthcare professionals, designed to elicit detailed information about each participant's medical history, severity and duration of HG symptoms, and previous pregnancies. Health records were reviewed to confirm diagnoses and gather additional clinical data. Diagnostic testing for H. pylori infection was performed using validated methods such as the urea breath test or stool antigen test, ensuring reliable confirmation of infection status. Participants were also subjected to clinical assessments to evaluate the severity of HG symptoms, including the frequency and intensity of nausea and vomiting episodes. In addition to clinical and diagnostic data, socioeconomic status was assessed through questionnaires that captured information on education level, employment status, income, and living conditions. This data was crucial for understanding potential socio-economic disparities that might influence both H. pylori infection rates and HG severity. Environmental factors such as housing density, sanitation conditions, and proximity to healthcare facilities were recorded through site visits and municipal records. All collected data were anonymized to protect participant confidentiality and securely stored in encrypted databases. Rigorous quality control measures were implemented to minimize data entry errors and ensure the reliability and validity of the dataset used for analysis.

Statistical analysis

Data were analyzed using SPSS version 25 (IBM Corp., Armonk, NY, USA). Descriptive statistics summarized baseline characteristics, including age, gestational age, and severity of HG symptoms. The eradication rate was calculated as the proportion of successfully treated patients using the urea breath test results. Comparative analyses were performed to evaluate changes in hospitalization duration, symptom severity, and need for intravenous fluids before and after treatment. Chi-square tests were used for categorical variables (e.g., presence of adverse effects), while paired t-tests were employed for continuous variables (e.g., hospitalization duration). A p-value of <0.05 was considered statistically significant, indicating that the observed differences were unlikely due to chance.

## Results

A total of 186 pregnant women were included in the study. The mean age of participants was 28.4 ± 5.6 years, and the average gestational age at enrollment was 12.3 ± 3.2 weeks, indicating that most women were in the first trimester when symptoms were most severe. The H. pylori eradication rate was 85.0% (158 patients), demonstrating high treatment efficacy. Correspondingly, 78.0% (145 patients) reported significant symptom improvement, while 22.0% (41 patients) experienced little or no relief. The mean duration of hospitalization was notably reduced from 7 ± 2 days before treatment to 3 ± 1 days after therapy, reflecting faster recovery and improved clinical stability post-eradication. Additionally, 60.2% of patients required intravenous fluids during hospitalization, while 39.8% managed without IV support (Table 2).

**Table 2:** Demographic Characteristics of Participants

Parameter	Value (n, %) / Mean ± SD
Total Participants	186 (100%)
Average Age (years)	28.4 ± 5.6
Gestational Age (weeks)	12.3 ± 3.2
Successful *H. pylori* Eradication	158 (85.0%)
Symptomatic Improvement	145 (78.0%)
No Improvement	41 (22.0%)
Average Hospitalization Duration Before Treatment	7 ± 2 days
Average Hospitalization Duration After Treatment	3 ± 1 days
Required IV Fluids	112 (60.2%)
Did Not Require IV Fluids	74 (39.8%)

The maternal and fetal outcomes following antibiotic therapy were largely favorable. The mean birth weight of newborns was 3200 ± 500 grams, indicating that fetal growth remained within the normal range. The mean gestational age at delivery was 39 ± 1.5 weeks, reflecting that most pregnancies progressed to full term. Only nine women (4.8%) experienced preterm deliveries, while seven infants (3.8%) were born low birth weight, both of which are relatively low frequencies and comparable to national averages. Regarding treatment safety, most participants (154 women, 82.8%) experienced no adverse effects, indicating that the antibiotic regimen was well tolerated. Among the few who reported side effects, minor gastrointestinal disturbance was the most frequent, occurring in 19 patients (10.2%), followed by headache in nine women (4.8%) and skin rash in four women (2.2%) (Table 3).

**Table 3:** Maternal, Fetal, and Safety Outcomes

Parameter	Value (n, %) / Mean ± SD
Average Birth Weight (grams)	3200 ± 500
Gestational Age at Delivery	39 ± 1.5 weeks
Preterm Deliveries	9 (4.8%)
Low Birth Weight Infants	7 (3.8%)
Mild Gastrointestinal Discomfort	19 (10.2%)
Headache	9 (4.8%)
Rash	4 (2.2%)
No Adverse Effects	154 (82.8%)

The average hospitalization duration before treatment was 7 ± 2.0 days, which significantly decreased to 3 ± 1 days after treatment. This reduction was statistically significant, with a p-value of <0.01, indicating that the improvement was unlikely due to chance (Table 4).

**Table 4:** Hospitalization Duration Before and After Treatment

Time Point	Mean ± SD (days)	t-value	df	p-value	Effect Size (Cohen’s d)
Before Treatment	7 ± 2				
After Treatment	3 ± 1	25.73	185	<0.001	1.89

## Discussion

This study shows an interesting result that validates the safety and outcomes of antibiotic treatment to eliminate H. pylori infection in pregnant patients with hyperemesis gravidarum (HG). The eradication rate of 85% demonstrated the standardized antibiotic regimen as an effective tool in eradicating a bacterial infection, which is one of the major contributors to HG symptoms. Moreover, symptom improvement in 78% of patients observed in this study speaks to the possibilities of H. pylori eradication as a possible treatment method to cope with HG more efficiently.

The eradication percentage realized in the current study is consistent with the literature regarding the success of combination antibiotic therapies in the non-pregnant population. Amoxicillin-Clarithromycin and a proton pump inhibitor (PPI) have been significantly reported in the synergistic effect in eradicating H. pylori. When it comes to the treatment of H. pylori in pregnancies, the antibiotics used have to strike the fine line between effectiveness and safety, therefore, not jeopardizing the growing fetus. The high rate of eradication (85%) in this study implies that the regimen chosen has managed to strike a balance between effective treatment and not affecting maternal or fetal well-being. The clinical advantage of efficient eradication of H. pylori is seen in the reduction of the length of stay in the hospital, which dropped by almost half (average of seven days of stay reduced to three days of stay following treatment). Lower hospitalization rates also help decrease stress on medical facilities, besides making patients more comfortable and satisfied with their stay. Such a decrease implies that the control of H. pylori infection could result in a faster stabilization of HG symptoms with a consequent decrease in the necessity of long-term medical treatment and supportive care, including the administration of intravenous fluids. The most important thing in the administration of any therapy in pregnancy is safety. It is relieving that there were no serious adverse effects in this research method, and it is thus revealed that the antibiotic regimen is well-received among pregnant women. It has some minimal side effects that include mild gastrointestinal discomfort (10%), headaches (5%) and rash (2%). These adverse effects were self-limiting and never required giving up of the therapy, a feature which clearly shows the safety of the regimen. The safety of the treatment is furthered by fetal outcomes. Seeing that their typical birth-weight was 3200 grams, and the gestational age at delivery was 39 weeks, the infants of mothers treated were within normal standards, about the same as national averages. The preterm deliveries (5%) and low birth weight infant rates (4%) were very low, which means that antibiotic therapy did not have any adverse impact on fetal development and pregnancy outcome [9,10].

Testing for H. pylori in HG

It may be possible to include H. pylori testing as part of HG management by screening all pregnant women with HG on a routine basis and determining if they would be likely to benefit from eradication treatment. This may have an early symptom relief, thus reducing the severity of HG and enhancing maternal health.

Standardized treatment protocols

To address the issue, it is important to develop and implement standardized treatment protocols that include the use of antibiotics that are developed for the needs of pregnant individuals. The use of protocols must take into account the trend of antibiotic resistance, the tolerability of patients, and the safety of the fetus to enhance optimal treatment.

Minimized hospitalization and healthcare costs

Proper treatment of symptoms attributed to H. pylori eradication comes with the possibility of decreased length of stay in hospitals and elimination of the intense care solutions like intravenous fluids. Such a decrease not only enhances patient experience but also removes the financial burden on healthcare systems. Mitigation of severe HG symptoms will help a great deal to increase the quality of life of pregnant mothers, and improve their nutritional intake, hydration, and physical as well as mental health throughout pregnancy [11,12].

Prior research recorded different efficacies and safety rates of antibiotic treatment in pregnant groups. For example, certain studies show lower rates of eradication because of the antibiotic resistance or treatment compliance differences. Nevertheless, the eradication rate observed in this study (85%) is comparable, not greater than the ones that are observed in the non-pregnant populations, which shows that both the regimen that has been chosen and its administration during pregnancy may be considered effective and acceptable. Besides, the safety outcomes conform with the findings in which some antibiotics, such as amoxicillin and clarithromycin, were usually safe to use during pregnancy, provided they are used properly. The low cases of adverse events also confirm the treatment regimen, hence its potential of becoming a standard treatment of HG among H. pylori-infected individuals [13,14]. Several studies bolster the hypothesis that Helicobacter pylori infection contributes to hyperemesis gravidarum. A meta-analysis of 38 studies by Ng et al. found a pooled odds ratio of 1.348, showing a statistically significant association between H. pylori positivity and HG [1]. In an Egyptian randomized controlled trial involving 156 HG patients with positive fecal antigen tests, women who received H. pylori eradication therapy (lansoprazole + amoxicillin) showed significantly greater symptomatic improvement than controls. The shorter the length of stay in hospitals, the less the healthcare expenses and efficient use of medical resources. Moreover, the low requirement of intensive schemes decreases the burden put on healthcare centers, especially in resource-constrained environments. The success level of eradication and symptomatic changes seems high, resulting in good compliance and adherence of the patient with the antibiotic regimen. It is essential that pregnant women be sensitized to take the full course of the antibiotic and, by so doing, enhance the effectiveness of the drugs as well as eliminate antibiotic resistance. To encourage patients to take care of themselves, hospitals and physicians could emphasize the importance of adherence to treatment plans, healthy lifestyle choices, and preventive care through structured education programs, visual aids, and personalized counseling [15,16].

Education of pregnant women on the possibility of H. pylori in causing HG and the positive effects of doing a treatment by eradication therapy is important in the decision-making process and treatment compliance. The effective provision of information should ensure that patients understand the safety and efficacy of the antibiotic regimen to actively take part in the creation of their treatment plan, which leads to establishing a collaborative healthcare culture. Antibiotic treatment of HG may not be accepted and conform to cultural beliefs and socioeconomic status. It is important to understand such factors in order to develop appropriate interventions that can address the needs of the various populations in an equal manner that promotes effective treatment. Mitigating factors inherent in the use of H. pylori eradication in control of HG must involve the tackling of barriers to the widespread adoption of the eradication strategy, which include the cost factor, accessibility of medications, and cultural stigmas that have been linked to the use of antibiotics. The findings of this study can be very pertinent to the global health scenarios, particularly in terms of the high prevalence of H. pylori and low accessibility to healthcare resources in areas with high prevalence rates of H. pylori infection. The burden of HG and its poor health outcomes, especially maternal and fetal, can be minimized specifically with effective yet affordable programs to initiate the eradication of H. pylori that can take additional pressure off overwhelmed healthcare systems in developing nations. Although this article dwelled on the contribution of H. pylori to HG, it is worth remembering the interaction of environmental and lifestyle factors that could affect the extent of H. pylori infection and HG. Various factors (including diet, hygiene, and exposure to environmental stressors) may influence transmission of H. pylori and colonization by the organism. Antibiotic therapy might be supplemented by comprehensive, public health-based responses that target these more upstream health determinants and thereby improve the overall treatment effect [17-19].

Limitations

More work needs to be done to understand the exact biological mechanism through which H. pylori plays a role in HG. The knowledge of interactions between the pathogen and the host, inflammatory signalling and the hormonal factors can guide the creation of pathogen-specific therapies and the transformation of treatment regimens. Further, the genetic components that can lead people to H. pylori infection and HG can be researched, as such a study will give a clue about personalized medicine in dealing with the condition. Limitations of the study are cross-sectional research and the absence of a control group. It is difficult to assess the improvement of the symptoms caused by antibiotic therapy alone without a comparison with either a placebo or some other treatment. Moreover, lack of long-term follow-up limits the evaluation of the curative effect and durability of maternal and pregnancy outcomes. The research was performed in one center as well, which can reduce the applicability of the results to other populations or settings. Subsequent research is needed to confirm these results, as well as to understand the long-term effects of the use of antibiotics during pregnancy by dividing a randomized controlled trial with a long follow-up time. Future clinical research can shift toward randomized controlled trials (RCTs), which involve treating people with antibiotic therapy and comparing it with a placebo or another therapy method to reveal more secure data about the efficacy and safety of antibiotic use. The long-term follow-up health outcomes of mothers and their children shall give an indication regarding the long-term effects of H. pylori eradication. Antibiotic resistance prevalence may be used to determine the choice of effective regimens due to antibiotic-resistant H. pylori prevalence in pregnant women.

## Conclusions

Antibiotic therapy for H. pylori eradication is a safe and effective treatment option for managing hyperemesis gravidarum in pregnant women. This approach not only alleviates severe symptoms but also enhances maternal and fetal health outcomes. By incorporating H. pylori testing and eradication into the management of HG, healthcare providers can offer a more comprehensive and effective treatment strategy, ultimately improving the quality of care for affected patients.
